# Harnessing metabolism of hepatic macrophages to aid liver regeneration

**DOI:** 10.1038/s41419-023-06066-7

**Published:** 2023-08-29

**Authors:** Rui Liu, Manuel Scimeca, Qiang Sun, Gerry Melino, Alessandro Mauriello, Changshun Shao, TOR Centre, Bernassola Francesca, Bove Pierluigi, Candi Eleonora, Rovella Valentina, Sica Giuseppe, Wang Ying, Yufang Shi, Mauro Piacentini, Giuseppe Tisone, Massimiliano Agostini

**Affiliations:** 1 Department of Experimental Medicine, TOR, University of Rome Tor Vergata, 00133 Rome, Italy; 2 Institute of Biotechnology, Academy of Military Medical Science; Research Unit of Cell Death Mechanism, 2021RU008, Chinese Academy of Medical Science, 100071 Beijing, China; 3 The First Affiliated Hospital of Soochow University, State Key Laboratory of Radiation Medicine and Protection, Institutes for Translational Medicine, Suzhou Medical College of Soochow University, 215123 Suzhou, Jiangsu China; 4 The First Affiliated Hospital of Soochow University, Institutes for Translational Medicine, State Key Laboratory of Radiation Medicine and Protection, Suzhou Medical College of Soochow University, 215123 Suzhou, China

**Keywords:** Mechanisms of disease, Cell death

## Abstract

Liver regeneration is a dynamic and regulated process that involves inflammation, granulation, and tissue remodeling. Hepatic macrophages, abundantly distributed in the liver, are essential components that actively participate in each step to orchestrate liver regeneration. In the homeostatic liver, resident macrophages (Kupffer cells) acquire a tolerogenic phenotype and contribute to immunological tolerance. Following toxicity-induced damage or physical resection, Kupffer cells as well as monocyte-derived macrophages can be activated and promote an inflammatory process that supports the survival and activation of hepatic myofibroblasts and thus promotes scar tissue formation. Subsequently, these macrophages, in turn, exhibit the anti-inflammatory effects critical to extracellular matrix remodeling during the resolution stage. However, continuous damage-induced chronic inflammation generally leads to hepatic macrophage dysfunction, which exacerbates hepatocellular injury and triggers further liver fibrosis and even cirrhosis. Emerging macrophage-targeting strategies have shown efficacy in both preclinical and clinical studies. Increasing evidence indicates that metabolic rewiring provides substrates for epigenetic modification, which endows monocytes/macrophages with prolonged “innate immune memory”. Therefore, it is reasonable to conceive novel therapeutic strategies for metabolically reprogramming macrophages and thus mediate a homeostatic or reparative process for hepatic inflammation management and liver regeneration.

## FACTS

- Hepatic macrophages play a key role in both hepatic homeostasis and liver regeneration.
- Hepatic macrophages acquire distinct metabolic phenotypes depending on their position in the liver lobule.
- Pro-inflammatory macrophages rely on aerobic glycolysis while anti-inflammatory macrophages rely on oxidative phosphorylation.
- Metabolic reprogramming of hepatic macrophages as a potential strategy for treating liver disease.

## OPEN QUESTIONS

- What are the long-term consequences of repolarizing macrophages towards an anti-inflammatory state?
- Concerning the heterogeneity of hepatic macrophages, will exogenous stimulation exert different effects on distinct macrophages?
- Is macrophage-based cell therapies an additional approach for treating liver disease?
- For macrophage-based cell therapy, are Kupffer cells or macrophages that are derived from other sources more suitable for treating liver disease?
- What is the best strategy for pre-treating macrophages in vitro?

## Introduction

The liver accounts for approximately 2% of the body weight in humans and among solid organs shows the highest regenerative capacity, and it is critical for a series of functions supporting digestion, metabolism, immunity, detoxification and vitamin storage [1, 2]. The high regenerative capacity of the liver [3] was originally described in rats undergoing partial hepatectomy, in which the original size of the liver was restored within several days of resection [4]. In humans, the liver can similarly regenerate to its normal size even after the removal of as much as 90% of total liver volume [5]. This powerful regenerative process involves the interaction of different cell types within sinusoids, including hepatocytes, biliary epithelial cells (cholangiocytes), endothelial cells, stellate cells and immune cells [6]. Moreover, hepatic macrophages play an important role in liver regeneration [7–11].

**Fig. 1:**
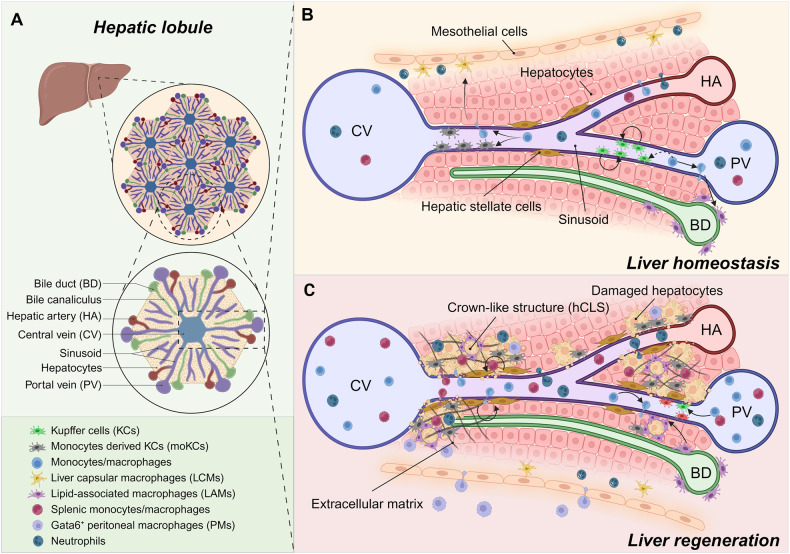
Spatiotemporal heterogeneity of hepatic macrophages. **A** The liver is composed of hexagonal lobules with several portal triads, including the bile duct, portal vein and hepatic artery, as well as a central vein. Hepatic macrophages that have either a fetal origin or have been derived from progenitors in the peripheral circulatory system play a critical role in maintaining liver homeostasis and promoting liver regeneration. **B** In the homeostatic liver, liver resident Kupffer cells (KCs) are distributed in periportal (PP) regions, where they are critical for capturing bacteria exiting the gut. These KCs show self-renewal capacity and acquire a tolerogenic phenotype, whereas macrophages located in the central vein region are characterized by a pro-inflammatory phenotype. Monocyte-derived liver capsular macrophages (LCMs) can sense and control intrahepatic bacterial dissemination by recruiting neutrophils to the capsule. KCs and LCMs occupy and defend two different pathogen entry points and mediate immune responses in the liver. Moreover, lipid-associated macrophages (LAMs) are near bile ducts and are activated by local lipid exposure. They are also derived from peripheral monocytes, which have been broadly associated with the immune response. **C** After liver injury, the number of KCs is markedly decreased, and they promote the recruitment of immune cells into the liver. In this context, the monocyte-derived KC (moKC) population contributes to replenishment of the quantity and functionality of the KC pool. Pro-inflammatory macrophages as well as circulatory splenic macrophages infiltrate the damaged liver and generate an inflammatory microenvironment, which activates hepatic stellate cells (HSCs) and induces the formation of scar tissue. Then, pro-inflammatory macrophages can be reprogrammed into repair-promoting phenotypes to promote liver regeneration. LAMs have also been reported to be required for hepatic regeneration by forming crown-like structures (hCLSs), and loss of hCLSs promotes liver fibrosis. Recently, Gata6^+^ peritoneal macrophages (PMs) have been shown to directly invade the liver through a non-vessel pathway and enhance liver repair.

The liver is a critical hub for regulating all metabolic processes in the body. At the microscopic level, the functional units of the liver comprise the innumerable lobules related to the portal triad (the hepatic artery, portal vein, and bile duct) and a central vein [12, 13] (Fig. 1A). Oxygenated blood from the hepatic artery mixes with nutrient-rich blood draining from the gut, and this mixture flows through the lobules via the sinusoidal network before draining into branches of the central vein. This spatial organization generates a series of metabolic gradients, such as an oxygen, a nutrient, and a waste product gradient, comprising an area of ‘metabolic zonation’ [6, 14]. Although whether hepatic macrophages located in different ‘metabolic zones’ depend on different metabolic pathways is unclear, anti-inflammatory hepatic macrophages rely on mitochondrial oxidative phosphorylation and contribute to liver repair and attenuation of liver fibrosis. In contrast, glycolysis-dependent macrophages exert proinflammatory effects to exacerbate liver damage [15, 16].

Here, we review the major metabolic pathways that control the distinct functions of hepatic macrophages in the homeostatic or damaged liver. We also discuss recent advances in therapeutic approaches that target the metabolism of hepatic macrophages in the context of liver regeneration.

## The origin and distribution of hepatic macrophages

Hepatic macrophages play crucial roles both in the maintenance of hepatic homeostasis and in the molecular mechanisms underlying liver disease [17, 18], and these functions involve the p53 network [19–27] or non-coding RNA [28–31] Indeed, the heterogeneity of hepatic macrophages and involvement of different subsets of macrophages are crucial to the pathogenesis of liver diseases (Supplementary Table 1).

Hepatic macrophages that generically express CD64, F4/80 and MER proto-oncogene, tyrosine kinase (MerTK) are widely distributed at distinct locations around the liver [32]. They can be either embryonically seeded in the liver, exhibiting self-renewal capacity, or derived from peripheric monocytes and infiltrate the liver and differentiate into cells with distinct functional phenotypes in response to the microenvironment [33]. Owing to single-cell and spatial transcriptome studies, the spatiotemporal heterogeneity of hepatic macrophages has been observed in the liver, which has led scientists to rethink the contribution of macrophages in regulating liver homeostasis and regeneration, see Fig. 1B and C [34].

## Hepatic macrophage formation of immune zones

**Fig. 2:**
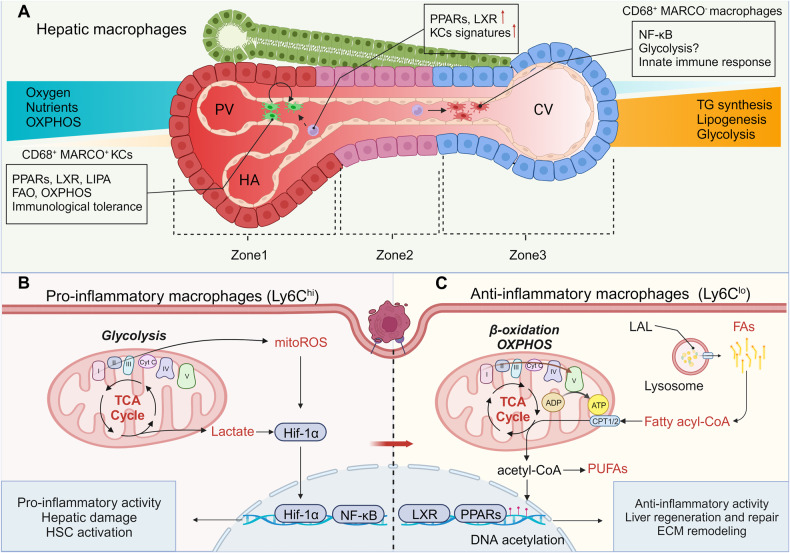
Macrophages in metabolic zones. Mitochondrial metabolism plays essential roles in macrophage function. Pro-inflammatory cytokines trigger glycolysis in macrophages and induce the immune response, while anti-inflammatory macrophages rely on oxidative phosphorylation (OXPHOS) and β-oxidation of fatty acids (FAO). **A** Metabolic zonation in the liver has been reported based on metabolic diversity in different regions of the liver. Blood circulation in the liver creates a series of gradients, such as oxygen, hormone, nutrient, and waste product gradients, which leads to enhanced OXPHOS and FAO in periportal regions and an increase in glycolysis around central veins. Accordingly, CD68^+^MARCO^+^ KCs distributed in periportal regions exhibit anti-inflammatory capacity. They express high PPARs and LIPA (encoding LAL), contributing to lipolysis and mitochondrial metabolism. LXR expression is also upregulated in these cells to regulate lipid metabolism and produce anti-inflammatory PUFAs. Notably, LXR is required for KC signature expression during the differentiation of monocytes into KCs. In contrast, CD68^+^MARCO^-^ macrophages are observed in central vein regions and are associated with inflammation. **B** During liver regeneration, KCs increase the uptake of glucose and trigger glycolytic metabolism. Ly6C^hi^ macrophages are recruited into the liver and accelerate inflammation. **C** Subsequently, they are further differentiated into resolutive Ly6C^lo^ macrophages to promote liver repair. Mitochondrial OXPHOS and β-oxidation of fatty acid occur in these macrophages, which contributes to the suppression of inflammation and ECM remodeling. The PPAR level is obviously increased in these cells compared with that in Ly6C^hi^ macrophages. This functional alteration is caused by phagocytosis. LAL lysosomal acid lipase, PUFA polyunsaturated fatty acid, PPARs peroxisome proliferator-activated receptors, LIPAs lipase A, LXR liver-x-receptor.

Hepatocytes display specialized functions in the area extending from the portal vein to the central vein, generating three distinct zones (Fig. 2A). Around the portal area (Zone 1), due to their proximity to oxygenated blood and nutrients, hepatocytes maintain oxidative metabolism that involves β-oxidation and amino acid catabolism as well as gluconeogenesis and bile and cholesterol formation [35]. With the oxygen gradient decreasing from the portal area to the central vein, hepatocytes that reside in Zone 3 are characterized by glycolytic metabolism, which is associated with a change in their function. Indeed, hepatocytes in Zone 3 participate in anabolism via lipogenesis, glycogen synthesis, and glutamine formation [6, 35].

Therefore, it is not surprising that hepatic macrophages also acquire distinct metabolic phenotypes depending on their position in the liver lobule [36, 37]. Single-cell RNA sequencing of the human liver revealed the characteristics of hepatic macrophages in different zones, as indicated by the gradient expression of the scavenger Macrophage receptor with collagenous structure (MARCO) [38]. MARCO is expressed only on noninflammatory Kupffer cells (KCs) that are accumulated in the periportal areas [38]. Human CD68^+^ MARCO^+^ cells seem to be at transcriptional level similar to long-lived liver resident mouse KC, whereas CD68^+^ MARCO^-^ macrophages exhibit a transcriptional profile similar to that of inflammatory recruited macrophages [39, 40].

## Metabolic alterations in hepatic macrophages

Macrophages exhibit extensive metabolic plasticity [41]. Specifically, a combination of a single-cell RNA-Seq, single-nucleus RNA-Seq, and spatial transcriptomics study of healthy human liver indicated that tolerogenic macrophage genes were present in periportal regions, whereas inflammatory macrophage genes were distributed around the central vein [42] (Fig. 2A). Pro-inflammatory macrophages rely on aerobic glycolysis associated with impaired oxidative phosphorylation (OXPHOS) (Fig. 2B), while anti-inflammatory macrophages rely on the tricarboxylic acid (TCA) cycle and enhanced mitochondrial oxidative metabolism [43] (Fig. 2C).

Mitochondria play pivotal roles in modulating macrophage function [44]. Indeed, macrophages stimulated with lipopolysaccharide (LPS) exhibited rewired metabolism, as indicated by decreased mitochondrial respiration, inhibition of the TCA cycle and upregulation of aerobic glycolysis. Although most of this metabolic reprogramming is controlled at the transcriptional level, there is evidence that metabolites may also regulate metabolic changes via inflammatory stimulus-induced activation [45]. For instance, the metabolite itaconate, which is highly produced in activated macrophages, regulates succination, mitochondrial respiration and cytokine production rates [46–48]. To support the involvement of mitochondria in regulating macrophage activation during tissue injury, it has recently been shown that the mitochondrial electron transport chain, particularly Complexes I, II and III, is also required for macrophage activation because they generate mitochondrial reactive oxygen species (mtROS) [49]. mtROS exert clear stabilization effects on the expression of hypoxia-inducible factor 1-alpha (HIF-1α), ultimately enhancing glycolysis and maintaining the function of the proinflammatory macrophage phenotype [43]. In contrast, alternative activation of macrophages has also been described during tissue repair, which is driven by oxidative metabolism fueled by enhanced consumption of fatty acids, glucose and glutamine. OXPHOS enhancement boosts the production of acetyl-CoA and activation of Jumonji domain-containing protein-3 (JMJD3), contributing to epigenetic modification of interleukin (IL)−4-inducible genes [43].

Macrophage differentiation is also sustained by fatty acid metabolism. LPS-challenged macrophages exhibit a disrupted TCA cycle and leads to an increase of cytosolic citrate which can be converted into acetyl-CoA, the building block of fatty acid synthesis, not consumed for fueling OXPHOS [50]. Hence, lipid synthesis is accepted as a mechanism that maintains the inflammatory phenotype of M1-like macrophages, whereas fatty acid oxidation (FAO) is required for M2-like macrophage polarization. However, recent evidence using macrophages with carnitine palmitoyltransferase (CPT)1A and CPT2 deficiencies suggested that FAO was not required for IL-4-induced macrophage polarization [51, 52], which demonstrated that the aforementioned mechanism is an oversimplified explanation for the role of lipid metabolism in macrophage function. Notably, SREBP1-mediated de novo lipogenesis is activated by LPS, which is also required for the resolution of inflammation caused by macrophages because it drives the production of anti-inflammatory fatty acids [53].

In the homeostatic liver, Kupffer cells (KCs) around portal regions exhibit tolerogenic capacity accompanied by reduced glycolysis, which seems to imply that they exhibit oxidative metabolism [54]. Accordingly, CD68^+^ MARCO^+^ noninflammatory KCs around portal regions exhibited upregulated expression of lipase A (LIPA) [38], a gene encoding the lysosomal acid lipase (LAL), which produces free fatty acids and cholesterol by hydrolyzing triglycerides and cholesteryl esters, which are required for FAO in M2 macrophages [55, 56] (Fig. 2A). On the other hand, CD68^+^ MARCO^-^ inflammatory macrophages activated nuclear factor kappa B (NF-κB) for glycolysis [57]. However, there is also evidence indicating a clear lipid metabolism signature of KCs when compared with the signature of other macrophage populations [58]. In particular, KCs express high levels of peroxisome proliferator-activated receptors gamma (PPARγ) and liver X receptor alpha (LXRα) as well as their related target genes implicated in the regulation of in lipid metabolism and cholesterol trafficking [59, 60]. PPARγ, a sensor of fatty acid-related genes [61], regulates fatty acid homeostasis in many cell types, which is also required for increased β-oxidation of fatty acids in anti-inflammatory macrophages [62]. Indeed, KCs deficient in PPARγ exhibit obvious impairment in alternative activation pathways and cause hepatic dysfunction [63].

LXR is a nuclear receptor that regulates triglyceride and cholesterol metabolism and is important to the anti-inflammatory functions of KCs [64]. LXR activation has been reported to promote polyunsaturated fatty acid synthesis in macrophages in a direct and sterol regulatory element-binding transcription factor 1 (SREBP1)-dependent manner [65]. Although the expression of SREBP1 can be activated by LPS to mediate de novo lipogenesis in pro-inflammatory macrophages, a recent study indicated that SREBP1 was also required for the resolution of inflammatory macrophages because it drives the production of fatty acid with of anti-inflammatory characteristic [53]. IL-4 can activate the SREBP1-mediated de novo lipogenesis program and thus support alternative macrophage activation pathway, suggesting a critical role for SREBP1 in maintaining the tolerogenic phenotype [66]. A recent study revealed that the expression KC signature genes, including Cd5 antigen-like (Cd5l), T cell immunoglobulin and mucin domain containing 4 (Timd4), Cd209l, procollagen C-endopeptidase enhancer 2 (Pcolce2), and placental associated 8 (Plac8), depend on LXR, and these genes are crucial for recruited monocytes to initiate and maintain KC identity [67]. This evidence indicates that LXR activation is required for KC homeostasis in the liver. In addition, large peritoneal macrophages (LPMs) exhibit a high oxidative phosphorylation rate fueled by glutamine and fatty acids in a steady state [68, 69]. LPMs are also very responsive to in vivo stimulation of IL-4 and, in addition, upregulate genes regulating OXPHOS and the TCA cycle, which possibly endow these cells with a repair-promoting phenotype in response to acute liver injury [70].

Liver injury and regeneration are dynamic processes involving inflammation and remodeling. After immunogenic activation, KCs enhance glucose uptake and pyruvate dehydrogenase kinase (PDK)-dependent glycolytic metabolism, which in turn reduces IL-10 production, affecting the tolerogenic potential of KCs [71]. Subsequently, Ly6C^hi^ monocyte-derived macrophages are recruited in a C-C Motif Chemokine Receptor 2 (CCR2)- and macrophage colony-stimulating factor (M-CSF)–mediated pathway during the necroinflammatory phase and then differentiate into an inflammation-resolving Ly6C^lo^ macrophage subset that expresses M2 genes and matrix metalloproteinases (MMPs) during the resolution phase [72]. Increasing evidence has demonstrated that arginine and fatty acids in apoptotic cells that have been engulfed can be reused by macrophages, inducing anti-inflammatory and polarization to the inflammation-resolving phenotype through subsequent polyamine metabolism and fatty acid oxidation, respectively [73, 74]. Accordingly, Ly6C^lo^ macrophages express more PPARγ target genes than macrophages expressing high levels of Ly6C, which indicates that mitochondrial metabolism may activated in inflammation-resolving macrophages. Depletion of this subpopulation in CD11b promoter–diphtheria toxin receptor (CD11B-DTR) transgenic mice led to failed scar remodeling and exacerbated fibrosis [75]. In response to a high-fat diet, binding with saturated fatty acids or entrapment of oxidized lipoproteins by macrophage scavenger receptor 1 (MSR1) led to the formation of foamy KCs with a proinflammatory phenotype [75].

Overall, although the metabolic heterogeneity of hepatic macrophages is still under investigation, mitochondrial oxidative metabolism is required for tolerogenic or inflammation-resolving macrophage polarization, which contributes to immunological tolerance or liver regeneration. On the other hand, in the early phase of liver damage, activated macrophages may trigger inflammatory responses that on glycolysis.

## Approaches to target macrophages to promote liver regeneration

**Fig. 3:**
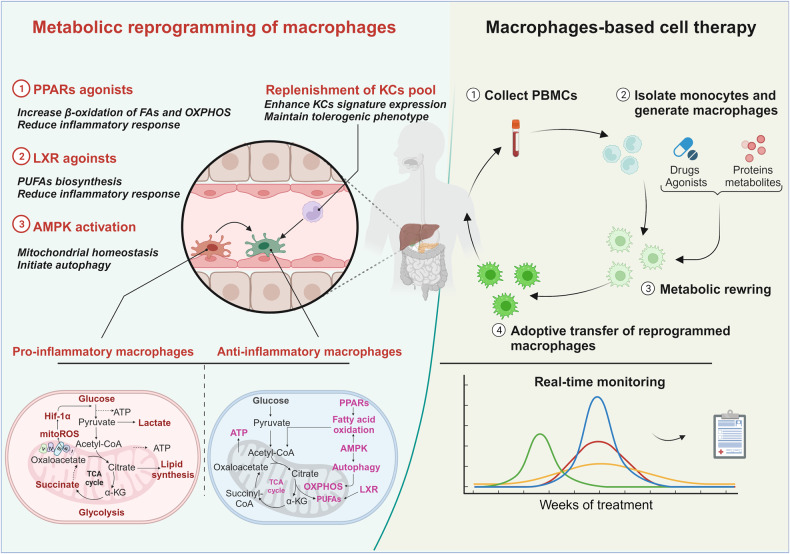
Therapeutic approaches for liver regeneration. Approaches targeting hepatic macrophages are shown. Metabolic reprogramming can be used to establish inflammation-resolving macrophages by reprogramming cells to undergo oxidative mitochondrial metabolism not proinflammatory glycolysis (left panel). An alternative strategy for boosting liver regeneration is macrophage-based cell therapy, which is being actively explored (right panel). See main text for details.

The modulatory properties of hepatic macrophages after liver damage and during damage repair make macrophages promising therapeutic targets for liver regeneration and liver disease treatment [76–78]. Recent strategies have been designed to reduce the recruitment or infiltration of monocytes/macrophages into the liver or to block the proinflammatory polarization of hepatic macrophages. However, several studies have demonstrated that inflammation-resolving macrophages mediate the remodeling of the extracellular matrix, which is required for liver regeneration. After inflammation-resolving macrophage polarization from pro-inflammatory macrophages fails, liver fibrosis is established. Considering the critical role of metabolism in modulating the functionality of macrophages, the metabolic reprogramming of hepatic macrophages is a potential therapeutic strategy that is being actively explored. A major goal of this novel therapeutic approach is to reverse liver fibrosis, turn off inflammation responses and trigger hepatocyte regeneration (Fig. 3).

### Metabolic reprogramming of hepatic macrophages

A strategy for metabolic rewiring of hepatic macrophages involves direct regulation of fatty acids β-oxidation and mitochondrial oxidative phosphorylation. PPARs constitute a family of transcription factors (α, β/δ, and γ) that regulate lipid and glucose metabolism and are expressed in homeostatic KCs [79]. Although PPARγ is expressed mainly in macrophages and induces β-oxidation of fatty acids, pan-PPAR agonists that target all PPAR isoforms have been reported to drive hepatic macrophages to acquire an anti-inflammatory phenotype and ameliorate liver fibrosis in experimental models of nonalcoholic steatohepatitis (NASH) (induced by diet) and chronic toxic injury (via chronic CCl_4_ administration) [80, 81]. In a mouse model of polycystic kidney disease fenofibrate treatment, a clinically available PPARα agonist, enhanced the β-oxidation rate of fatty acids and ameliorated liver disease [82]. Activation of PPARα and PPARβ/δ with elafibranor induced the hepatic expression of genes that regulates fatty acid β-oxidation including, Acyl-CoA dehydrogenase medium chain (Acadm) and Acyl-CoA oxidase 1 (Acox1) and potently reduced glycoprotein NMB (Gpnmb) expression on KCs, which was closely associated with liver injury and fibrosis in a cohort of mice with varying degrees of NASH severity [83]. Interestingly, a Phase 2b clinical trial (NCT03008070) involving 247 patients with active NASH showed that the percentage of patients who presented with a decrease in the SAF-A score (Steatosis, Activity, Fibrosis [SAF] scoring system) of at least 2 points without worsened fibrosis was significantly higher in patients treated with Lanifibranor than those treated with placebo [84].

In macrophages, PPARγ isoform exhibits anti-inflammatory features by downregulating the expression of inflammation-related gene. Macrophage-specific depletion of PPARγ exacerbated necroinflammatory injury [85]. The pharmacological treatment of rat KCs with a PPARγ agonist (pioglitazone hydrochloride) inhibited nitric oxide (NO) and tumor necrosis factor-alpha (TNF-α) induced by LPS. Another PPARγ agonist, thiazolidinediones, reversed hepatic insulin resistance in mice fed a high-fat diet [86, 87]. After depleting PPARγ in macrophages, the therapeutic effect of thiazolidinediones disappeared, suggesting that PPARγ was required for the modulatory effects of macrophages on the liver microenvironment [86] Clinically, patients with nonalcoholic fatty liver disease who received pioglitazone in a Phase 2 clinical trial (NCT00633282) showed reduced ALT and AST activities, which indicated that PPARγ activation effectively protected against liver damage. Interestingly, four weeks of preoperative exercise therapy induced anti-inflammatory-trained immunity in KCs mediated through metabolic reprogramming triggered by the metabolite itaconate [88]. Notably, PPARδ and adenosine 5’-monophosphate-activated protein kinase (AMPK) agonists have been shown to act as exercise mimetics [89].

An additional therapeutic target is the nuclear receptor LXRα, which is highly expressed in KCs [90]. LXR agonist treatment repressed the expression of pro-inflammatory inducible nitric oxide synthase (iNOS) and cyclooxygenase-2 (COX-2) in LPS-challenged macrophages by inhibiting the dissociation of a corepressor protein from nuclear factor-κB (NF-κB) [91–93]. LXR activation also suppressed Toll like receptor (TLR) ligand-dependent inflammatory effects mediated through an ATP-binding cassette transporter (ABCA1)-mediated pathway or direct modulation of the accessibility of proinflammatory gene enhancers in chromatin through a cis-repressing interaction [94, 95] LXRs also exerted anti-inflammatory effects by increasing omega-3 polyunsaturated fatty acid levels (PUFA), which primarily inhibited NF-kB-dependent inflammation in macrophages [96]. A pharmacologically induced increase in the LXR ligand desmosterol (a sterol intermediate in cholesterol synthesis) led to an increase in LXR signaling and PUFA biosynthesis, effectively skewing macrophage polarization towards the inflammation-resolving phenotype [97]. Nuclear hormone receptors are also implicated in the regulation of efferocytosis, a process by which apoptotic cells are eliminated and inflammation is suppressed. Engulfment of apoptotic cells by alternatively activated human macrophages led to an accumulation of sterol intermediates (desmosterol, lathosterol, lanosterol, and dihydrolanosterol) which in turn led to the activation of the LXR-dependent downstream pathway resulting in the increased expression of anti-inflammatory genes. The polarization of these cells into anti-inflammatory macrophages was also induced via treatment with the synthetic LXR agonist T0901317 [97].

These studies may partially reveal the mechanism by which peripheral monocytes/macrophages replenish the KC pool during liver regeneration. Although LXR agonists exert anti-inflammatory effects on macrophages, the development of LXR agonists for the pharmacological treatment of liver disease has proven quite challenging due to side effects, including hepatic steatosis and hypertriglyceridaemia [98]. Indeed, although several synthetic LXR agonists have been entered into Phase I clinical trials, none were evaluated further due in part to adverse effects.

AMPK is an intracellular energy sensor that can boost oxidative catabolism and autophagy by maintaining mitochondrial homeostasis, which ultimately promotes macrophage polarization into an anti-inflammatory phenotype [99]. Therefore, AMPK plays an important role in regulating several metabolic pathways and is involved in some human pathological conditions, including type 2 diabetes, non-alcoholic fatty liver disease, and cardiovascular disease [100]. In mice treated with CCl_4_ to induce fibrosis, ferulic acid (FA) administration ameliorated hepatic inflammation and subsequent fibrosis. FA directly binds to and inhibits protein tyrosine phosphatase 1B (PTP1B), an enzyme critical for dephosphorylating key protein kinases, eventually resulting in the phosphorylation of AMPK in macrophages. The antifibrotic effect of the AMPK activator HL156A has also been reported to block the activation of macrophages, subsequently reducing thioacetamide-induced liver fibrosis [101]. Preclinical data strongly suggested that AMPK-mediated autophagy is required for liver regeneration. In fact, autophagy Related 5 (Atg5) depletion in macrophages boosted proinflammatory IL1α and IL1β levels, which further aggravated liver injury and fibrosis [102]. Blocking autophagy with 3-methyladenine promoted KCs survival, which in turn further abrogated UCN-induced liver toxicity. Interestingly, reduced autophagy via increased NLRP3 inflammasome activity is also evident in the livers of patients with NASH [103].

Ezetimibe, a widely prescribed drug for treating hypercholesterolemia, boosted autophagic flux in an AMPK-dependent manner and concomitantly ameliorates lipid accumulation and inhibited apoptosis in palmitate-exposed hepatocytes [103]. In addition, spermidine, a natural polyamine that is derived from the catabolism of arginine, exhibited effective therapeutic potential for managing liver injury and fibrosis [104–106] Our previous study demonstrated that spermidine promotes mitochondrial metabolism by activating AMPK and autophagy in macrophages [107]. To date, many AMPK activators including berberine, AICAR, resveratrol, palmitoleate and A769662, which are also reviewed in detail elsewhere [108], have been shown to diminish macrophage-mediated inflammation, supporting these activators as potential candidates for promoting liver regeneration by targeting metabolic AMPK in macrophages.

### Macrophage-based cell therapies

The potential for macrophage-based cell therapies to inhibit inflammation and trigger orderly extracellular matrix remodeling is being actively investigated. The effects of syngeneic bone marrow-derived macrophages, their specific precursors from bone marrow or unfractionated full bone marrow were characterized in mice with CCl_4_-induced liver fibrosis [109]. In this experimental setting, only differentiated bone marrow-derived macrophages, not their precursors, were obviously able to attenuate fibrosis. In contrast, liver fibrosis was significantly exacerbated by whole bone marrow cells. These macrophages were injected via a peripheral vein homed to the liver, recruited host immune cells to generate liver scars via chemokine upregulation, released MMP-13 and 9 and increased the level of the anti-inflammatory cytokine IL-10, subsequently ameliorating liver fibrosis [109].

Thus, preclinical studies suggest paracrine activity mediated through transplanted macrophages not whole BMCs in the regenerative niche. A clinical trial is currently underway to evaluate the safety and feasibility of infusion of peripheral monocyte-derived macrophages isolated from patients with cirrhosis. An attempt to transfer human monocyte-derived macrophages from healthy donors or even donors with cirrhosis into mice with liver fibrosis may be effective in ameliorating liver fibrosis [110]. Interestingly, macrophages from not only donors but also patients with cirrhosis display a similar phenotype, which indicates the potential for autologous macrophage therapy for liver fibrosis or cirrhosis. Thus, autologous monocyte-derived macrophages were tested in a first-in-human, Phase1, single-arm, dose-escalation clinical trial with patients with cirrhosis (ISRCTN 10368050). The primary outcomes for safety and feasibility are met, without adverse events recorded after injection, macrophage activation syndrome or dose-dependent toxicity. Notably, a decrease in indicators associated with liver fibrosis was found in most patients who received the macrophage infusion [110].

Given the functional variability of hepatic macrophages in orchestrating liver regeneration, polarized macrophages have also been considered potential cell therapeutic candidates in preclinical models of acute and chronic liver injury. Transfer of proinflammatory macrophages treated with LPS and IFN-γ in vitro rather than IL-4 to activate anti-inflammatory or unpolarized macrophages effectively ameliorated liver fibrosis by modifying the recruitment and activation of endogenous macrophages and natural killer cells [111].

In addition, recent evidence has revealed that alternatively activated macrophages (AAMs) are primarily Ly6C^lo^ cells and exhibit remarkable phagocytic capability in preclinical models of acetaminophen overdose. Adoptive transfer of human AAMs markedly elicited a reduction in hepatic necrosis and boosted the proliferation of hepatocytes and endothelial cells in mice with liver damage [112]. These studies possibly indicate that distinct polarized macrophages may be required at different stages of liver regeneration.

Therefore, transplantation of ex vivo metabolically reprogrammed macrophages may be an additional strategy for the use of anti-inflammatory macrophages. It is reasonable to think that polarized macrophage-based therapies with macrophages treated only with LXR agonists may prevent hepatic lipogenesis.

## Conclusions and perspectives

Owing to increasing numbers of single-cell and spatial transcriptomic studies, the origin, functional and metabolic diversities of hepatic macrophages have been highlighted in both hepatic homeostasis and liver regeneration contexts. In general, hepatic macrophages, especially KCs that express PPAR and LXR, exhibit mainly a tolerogenic phenotype and contribute to immunological tolerance with reliance on mitochondrial oxidative metabolism in the homeostatic liver. PPAR and LXR are required for the differentiation and functionality of KCs. Once the liver is damaged, KCs can be activated through the glycolytic pathway and thus promote the recruitment of peripheral Ly6C^hi^ monocytes/macrophages, which make up the inflammatory microenvironment. Their recruitment leads to excessive deposition of extracellular matrix components produced by activated hepatic myofibroblasts and to subsequent liver fibrosis. Notably, macrophages can be converted into an inflammation-resolving phenotype manifested by upregulated PPAR, anti-inflammatory genes and metalloproteinase protein expression after phagocytosis, which is required for microenvironmental remodeling. Thus, metabolic rewiring of hepatic macrophages is increasingly regarded as a potential strategy for treating liver disease.

**Fig. 4:**
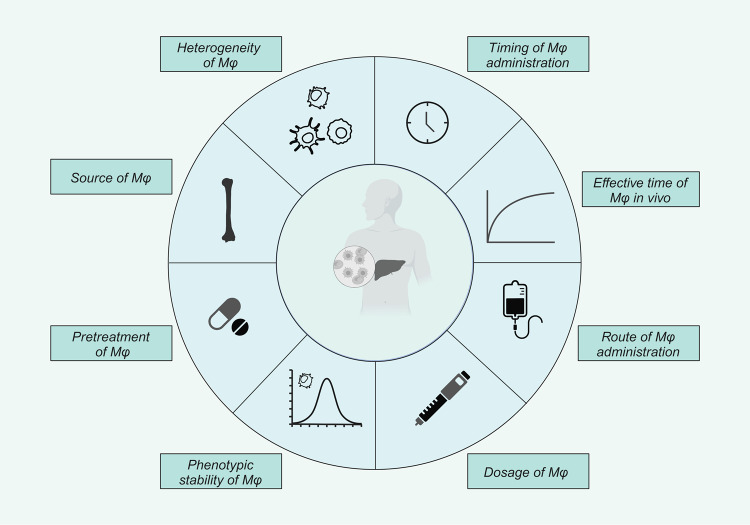
Challenges of hepatic macrophage-targeted strategies. Many key parameters need to be optimized in macrophage-based therapy designs. These parameters involve the sources and heterogeneity of macrophages, and an optimized protocol for macrophage pre-treatment, stability of macrophage function, dosage, are routes and timing for macrophage injection, which are crucial for the successful application of macrophages in clinical settings, need to be identified. Mϕ: macrophages.

Recent preclinical and clinical progress in the field of liver regeneration have also indicated the promising approach of macrophage-based cell therapy to treat acute and chronic liver disease [34, 113]. To expedite the translation from bench to bedside, several challenges must be overcome, and requirements must be met for the success of hepatic macrophage-targeted approaches. Many questions remain to be answered (Fig. 4). How can the dual role of metabolic agonists, such as LXR agonists, between macrophages and hepatocytes be prevented? What are the long-term consequences of repolarizing macrophages towards an anti-inflammatory state? More importantly, concerning the heterogeneity of hepatic macrophages, will exogenous stimulation exert different effects on distinct macrophages? For macrophage-based cell therapy, are KCs or macrophages that are derived from other sources more suitable for treating liver disease? What is the best strategy for pre-treating macrophages in vitro? In addition, an optimized protocol for inducing stable macrophage polarization as well as determining the dose, timing and route of macrophage administration need also be confirmed through carefully designed clinical trials.

## Supplementary information

Supplementary Materials
