# Supplementary material for: Harnessing metabolism of hepatic macrophages to aid liver regeneration
Source: Cell Death Dis. 2023 Aug 29;14(8):574. doi: 10.1038/s41419-023-06066-7 (PMC10465526; doi:10.1038/s41419-023-06066-7)
Supplement: Supplementary file 1 — Supplementary Materials [file 41419_2023_6066_MOESM1_ESM.pdf]

Supplementary table 1. Hepatic macrophage subsets

Kupffer Cells (KCs)		Liver capsular macrophages (LCMs)	Lipid-associated macrophages (LAMs)	Peripheral macrophages (PMs)	Splenic macrophages (sMs)			Peritoneal macrophages (PMs)	
Conserved kupffer cells (KCs)	Monocytes-derived Kupffer Cells (moKCs)				Splenic monocytes-1 (sM-1s)	Splenic monocytes-2 (sM-2s)	Splenic macrophages (sMs)	Large peritoneal macrophages (LPMs)	Small peritoneal macrophages (SPMs)
F4/80	F4/80	F4/80	F4/80	CD11b	CD11b	CLEC4D	F4/80	F4/80^{hi}	F4/80^{low}
CD64	CD64	CD64	CD64	F4/80	CD43^{hi}	MSR1	MHC-II	CD11b^{hi}	CD11b^{low}
CLEC4F	CLEC2 (early)	MHCII	GPNMB	Ly6C^{hi} (Pro-inflammatory)	Ly6C^{low}	CSTB	CD64	GATA6	MHC-II^{hi}
CLEC2	CLEC4F	CD11c	SPP1	Ly6C^{low} (Anti-inflammatory)		MIF		CD206	Ly6C
TIM4	CD5L	CD14	TERM2					CD64	
CD163	VSIG4	CSF1R	CD9					CD68	
CD5L	FOLR2	CX_3CR1	CX_3CR1					CD11c	
VSIG4	MARCO							CD115	
FOLR2	F4/80							CD102	
MARCO	SLC40A1								
F4/80									
SLC40A1									
Reference: 1, 2, 3, 4, 5, 6, 7, 8, 9	1, 2, 3, 4, 5, 6, 7, 8, 9	9, 10, 11, 12	13, 4, 9, 14, 15	16, 17, 18		19, 20, 21, 22		23, 24, 25, 26	

Reference:

1) Guilliams, M. & Scott, C. L. Liver macrophages in health and disease. *Immunity* **55**, 1515-1529, doi:10.1016/j.immuni.2022.08.002 (2022).
2) Scott, C. L. *et al.* Bone marrow-derived monocytes give rise to self-renewing and fully differentiated Kupffer cells. *Nat Commun* **7**, 10321, doi:10.1038/ncomms10321 (2016).
3) Beattie, L. *et al.* Bone marrow-derived and resident liver macrophages display unique transcriptomic signatures but similar biological functions. *J Hepatol* **65**, 758-768, doi:10.1016/j.jhep.2016.05.037 (2016).
4) Remmerie, A. *et al.* Osteopontin Expression Identifies a Subset of Recruited Macrophages Distinct from Kupffer Cells in the Fatty Liver. *Immunity* **53**, 641-657 e614, doi:10.1016/j.immuni.2020.08.004 (2020).
5) Sakai, M. *et al.* Liver-Derived Signals Sequentially Reprogram Myeloid Enhancers to Initiate and Maintain Kupffer Cell Identity. *Immunity* **51**, 655-670 e658, doi:10.1016/j.immuni.2019.09.002 (2019).
6) Scott, C. L. *et al.* The Transcription Factor ZEB2 Is Required to Maintain the Tissue-Specific Identities of Macrophages. *Immunity* **49**, 312-325 e315, doi:10.1016/j.immuni.2018.07.004 (2018).
7) Pallett, L. J. *et al.* Longevity and replenishment of human liver-resident memory T cells and mononuclear phagocytes. *J Exp Med* **217**, doi:10.1084/jem.20200050 (2020).
8) Tran, S. *et al.* Impaired Kupffer Cell Self-Renewal Alters the Liver Response to Lipid Overload during Non-alcoholic Steatohepatitis. *Immunity* **53**, 627-640 e625, doi:10.1016/j.immuni.2020.06.003 (2020).
9) Guilliams, M. *et al.* Spatial proteogenomics reveals distinct and evolutionarily conserved hepatic macrophage niches. *Cell* **185**, 379-396 e338, doi:10.1016/j.cell.2021.12.018 (2022).
10) Sierro, F. *et al.* A Liver Capsular Network of Monocyte-Derived Macrophages Restricts Hepatic Dissemination of Intraperitoneal Bacteria by Neutrophil Recruitment. *Immunity* **47**, 374-388 e376, doi:10.1016/j.immuni.2017.07.018 (2017).
11) Balog, S. *et al.* Development of Capsular Fibrosis Beneath the Liver Surface in Humans and Mice. *Hepatology* **71**, 291-305, doi:10.1002/hep.30809 (2020).
12) Bleriot, C. & Ginhoux, F. Understanding the Heterogeneity of Resident Liver Macrophages. *Front Immunol* **10**, 2694, doi:10.3389/fimmu.2019.02694 (2019).
13) Daemen, S. *et al.* Dynamic Shifts in the Composition of Resident and Recruited Macrophages Influence Tissue Remodeling in NASH. *Cell Rep* **34**, 108626, doi:10.1016/j.celrep.2020.108626 (2021).
14) Jaitin, D. A. *et al.* Lipid-Associated Macrophages Control Metabolic Homeostasis in a Trem2-Dependent Manner. *Cell* **178**, 686-698 e614, doi:10.1016/j.cell.2019.05.054 (2019).

15) Ramachandran, P. *et al.* Resolving the fibrotic niche of human liver cirrhosis at single-cell level. *Nature* **575**, 512-518, doi:10.1038/s41586-019-1631-3 (2019).
16) Cheng, D. *et al.* Hepatic macrophages: Key players in the development and progression of liver fibrosis. *Liver Int* **41**, 2279-2294, doi:10.1111/liv.14940 (2021).
17) Fogg, D. K. *et al.* A clonogenic bone marrow progenitor specific for macrophages and dendritic cells. *Science* **311**, 83-87, doi:10.1126/science.1117729 (2006).
18) Ramachandran, P. *et al.* Differential Ly-6C expression identifies the recruited macrophage phenotype, which orchestrates the regression of murine liver fibrosis. *Proc Natl Acad Sci U S A* **109**, E3186-3195, doi:10.1073/pnas.1119964109 (2012).
19) Wu, W., Zhang, J., Yang, W., Hu, B. & Fallon, M. B. Role of splenic reservoir monocytes in pulmonary vascular monocyte accumulation in experimental hepatopulmonary syndrome. *J Gastroenterol Hepatol* **31**, 1888-1894, doi:10.1111/jgh.13388 (2016).
20) Swirski, F. K. *et al.* Identification of splenic reservoir monocytes and their deployment to inflammatory sites. *Science* **325**, 612-616, doi:10.1126/science.1175202 (2009).
21) Zhang, S. *et al.* CD11b(+) CD43(hi) Ly6C(lo) splenocyte-derived macrophages exacerbate liver fibrosis via spleen-liver axis. *Hepatology*, doi:10.1002/hep.32782 (2022).
22) Li, L. *et al.* The Spleen Promotes the Secretion of CCL2 and Supports an M1 Dominant Phenotype in Hepatic Macrophages During Liver Fibrosis. *Cell Physiol Biochem* **51**, 557-574, doi:10.1159/000495276 (2018).
23) Gautier, E. L. *et al.* Gata6 regulates aspartoacylase expression in resident peritoneal macrophages and controls their survival. *J Exp Med* **211**, 1525-1531, doi:10.1084/jem.20140570 (2014).
24) Ghosn, E. E. *et al.* Two physically, functionally, and developmentally distinct peritoneal macrophage subsets. *Proc Natl Acad Sci U S A* **107**, 2568-2573, doi:10.1073/pnas.0915000107 (2010).
25) Kierdorf, K., Prinz, M., Geissmann, F. & Gomez Perdiguero, E. Development and function of tissue resident macrophages in mice. *Semin Immunol* **27**, 369-378, doi:10.1016/j.smim.2016.03.017 (2015).
26) Cassado Ados, A., D'Imperio Lima, M. R. & Bortoluci, K. R. Revisiting mouse peritoneal macrophages: heterogeneity, development, and function. *Front Immunol* **6**, 225, doi:10.3389/fimmu.2015.00225 (2015).
